# The Effectiveness of the Turkish Version of the Responsive Teaching Program Applied as an Online Group Intervention on Autistic Children and Their Fathers: A Randomized Control Study

**DOI:** 10.3390/bs15030309

**Published:** 2025-03-05

**Authors:** Ali Kaymak, Ibrahim H. Diken, Gerald Mahoney

**Affiliations:** 1 Research Institute for Individuals with Developmental Disabilities, Anadolu University, 26470 Tepebaşı, Turkey; alikaymak@anadolu.edu.tr; 2 Jack, Joseph and Morton Mandel School of Applied Social Sciences, Case Western Research University, Cleveland, OH 44106, USA; gerald.mahoney@case.edu

**Keywords:** autistic children, Turkish version, responsive teaching (RT) program, fathers, online training

## Abstract

The purpose of this study was to explore the effectiveness of the Turkish Version of the Responsive Teaching program applied as an online group intervention on autistic children and their fathers. In this study, conducted with pre-test–post-test control group experimental design, 20 father–child pairs were randomly assigned to the experimental and control groups. This study’s independent variable was the implementation of the Turkish Version of the Responsive Teaching (TV-RT) program (TV-RT) applied as an online group intervention. The dependent variables of this study were (a) fathers’ interactional behaviors, (b) fathers’ ability to use TV-RT strategies, (c) children’s interactional behaviors, (d) children’s ability to use TV-RT pivotal behaviors, (e) children’s social interaction behaviors (typical social interaction and autistic interaction), and (f) the opinions of the fathers and mothers of the children in the experimental group about the program and results. Data were collected with the Turkish Version of the Maternal/Parent Behavior Rating Scale (M/PBRS-TV) to measure fathers’ interactional behaviors; the Responsive Teaching–Parent Strategy Profile (RT-PSP) to measure fathers’ level of use of the TV-RT strategies; the Turkish Version of the Child Behavior Rating Scale (CBRS-TV) to measure children’s interactional behaviors; the Responsive Teaching–Pivotal Behavior Profile (RT-PBP) to measure children’s level of displaying TV-RT pivotal behaviors; and the Turkish Version of the Social Interaction Assessment Instrument (SIAI-TV) to measure children’s social interaction behaviors (typical social interaction and autistic interaction). In addition, social validity data were collected from the fathers and mothers in the experimental group through satisfaction questionnaires. Results revealed that the fathers of autistic children who received the intervention differed significantly on the use of TV-RT strategies and the quality of interactional behaviors from the fathers who did not receive the intervention. On the other side, autistic children in the experimental group showed significant progress on interactional behaviors, TV-RT pivotal behaviors, and social interaction behaviors and improved changes on autistic interaction behaviors. Fathers who participated in the online group TV-RT program, and their wives (mothers) as outside observers, reported high satisfaction with the program. Results were discussed extensively, and future suggestions are provided.

## 1. Introduction

Early childhood education is crucial, as delaying investment in children’s development can lead to significant social and economic costs. By stating that *“We don’t have the luxury of waiting to invest in children until they start primary school. Because then it may be too late to intervene”.* Heckman (2011), awarded the Nobel Prize for his socio-economic studies on the economic return of early childhood education, emphasizes that early intervention benefits disadvantaged children, reducing the need for special education, improving health outcomes, decreasing crime rates, and lowering overall social costs. Children require interactive experiences to understand their environment (Davis & Scherz, 2022; Eagleman, 2020), supported by an innate developmental structure (Einon, 1998) and their immediate environment, particularly parents (Bronfenbrenner, 1979). Various factors, including socio-economic status, parenting styles, and available stimuli, shape a child’s development (García-Ventura et al., 2023). Parents’ involvement in early interventions is especially critical for children with special needs (Blacher et al., 2013). Research shows that these parents often struggle with effective interactions due to stress, anxiety, and a lack of knowledge (Baker-Ericzén et al., 2005; Doubet, 2012; Falk et al., 2014; Gau et al., 2012; Ostfeld-Etzion et al., 2016; Ozturk et al., 2014; Tomris & Diken, 2021). These challenges can result in decreased engagement with the child, hindering the development of skills such as joint attention, imitation, and language acquisition (Dracobly & Smith, 2022; Nevill et al., 2018; Pierce et al., 2023; Zwaigenbaum et al., 2013).

Autism Spectrum Disorder (ASD) is one of the most frequently diagnosed neurodevelopmental conditions requiring early intervention (Maenner et al., 2023). Social communication difficulties, a key diagnostic criterion, emerge before one year of age and intensify without intervention (Zwaigenbaum et al., 2013). Autistic children often struggle with eye contact and social responsiveness (Dawson et al., 2023; McKenzie & Dallos, 2017), affecting their ability to initiate and maintain interactions (Landa, 2018; Pierce et al., 2023). These difficulties reduce their inclination to engage with caregivers (Nevill et al., 2018), while parents also face challenges in fostering meaningful interactions. Additionally, difficulties in expressive and receptive language can lead to frustration and behavioral issues (Matson & Adams, 2014), increasing parental stress (Joyce et al., 2017). Without timely intervention, these challenges worsen over time (Dracobly & Smith, 2022). Early intervention, leveraging neural plasticity, significantly improves developmental outcomes (Davis & Scherz, 2022; Eagleman, 2020). Nahmias et al. (2019) and L. K. Koegel et al. (2014) highlight that early diagnosis and intervention benefit autistic individuals, their families, and society. Piccininni et al. (2017) found that reducing the average nine-month waiting time for early intervention could save USD 267,000 per individual.

Several intervention approaches exist for autistic children (Rogers & Vismara, 2008). Some emphasize structured, school-based training for parents (Lovaas, 1987), while others advocate naturalistic, relationship-based approaches that integrate learning into daily life (Mahoney & Powell, 1988). Family-centered interventions (FCIs), which empower parents and prioritize quality interactions, have demonstrated effectiveness (Mahoney & Perales, 2011). Engaging parents in early intervention enhances efficiency, cost-effectiveness, and the generalization of skills (Akemoğlu & Meadan, 2018; Cheng et al., 2023; Lord et al., 2022; Nahmias et al., 2019; Rogge & Janssen, 2019; Zwaigenbaum et al., 2015). Given the evidence supporting family involvement, FCIs are now widely recognized as best practices (Bakkaloğlu, 2020). Responsive Teaching (RT), developed by Mahoney, is a family-centered approach that addresses parent–child interactions. RT strategies, such as following a child’s lead, taking turns in communication, and using engaging commentary, have been linked to improved communication and cognitive and social development (Ö. Diken & Mahoney, 2013; J.-M. Kim & Mahoney, 2004; Mahoney & Perales, 2003). However, research indicates that parents of autistic children often adopt a directive and less responsive approach, necessitating additional support (Wan et al., 2012; Töret et al., 2015). RT strategies have been recommended to enhance interaction quality, particularly for fathers (Oğuz & Sönmez, 2017).

RT became a structured family-centered program in 2006 and was adapted into Turkish as ETEÇOM in 2010. While studies on RT have been conducted globally (Ö. Diken & Mahoney, 2013; Karaaslan et al., 2013; Karaaslan, 2016; J.-M. Kim & Mahoney, 2004; Mahoney, 2009; Mahoney & Perales, 2003, 2005; Toper et al., 2019), fathers have rarely been the focus. The limited participation of fathers in early intervention is not unique to ETEÇOM, as studies on father–child interactions in ASD remain scarce (Elder et al., 2011). Marsack-Topolewski (2023) found that 98% of studies involving parents of autistic children did not specifically examine fathers. Similarly, Orum Çattık et al. (2020) found that no studies solely focused on fathers in early childhood interventions. This lack of research persists, despite calls for a greater emphasis on fathers’ roles in intervention (Panter-Brick et al., 2014; Rankin et al., 2019; Stahmer & Pellecchia, 2015; Tiano & McNeil, 2005). Fathers face barriers to participation, including societal expectations, work schedules, and time constraints (Bozok, 2018; Rivard et al., 2014; Johnson & Simpson, 2013; Gonzalez et al., 2023). One potential solution is online interventions, which offer flexibility and accessibility (Akemoğlu et al., 2022). Digital interventions, particularly those incorporating parent coaching, have been shown to enhance efficiency while reducing costs (Akemoğlu & Meadan, 2018; Douglas et al., 2020; Lee et al., 2022; Garnett et al., 2022; Sawyer & Campbell, 2017; Sone et al., 2021; Tanner & Dounavi, 2020; Vismara et al., 2018).

Barriers to father participation include social norms, professional attitudes, travel costs, and scheduling conflicts (Gonzalez et al., 2023; McWayne et al., 2013; Rankin et al., 2019). Telehealth interventions offer a promising alternative (Piotrowska et al., 2020). Telehealth involves using video conferencing and other communication technologies to provide professional support remotely (R. L. Koegel et al., 2002). Research on telehealth interventions for parents of autistic children suggests that they are as effective as, if not superior to, in-person services, in terms of time and cost efficiency (Crone & Mehta, 2016; Jang et al., 2012; Koonin et al., 2020). Furthermore, telehealth can enhance engagement and motivation among fathers who face barriers to participation. Studies show that telehealth interventions improve parenting skills and enhance children’s language and social development (Dahiya et al., 2022; Gerow et al., 2021; Ingersoll et al., 2016; Jang et al., 2012; Vismara et al., 2018). The benefits of telehealth-based parent training, such as reduced costs and increased accessibility, have generated optimism for its widespread use (Sadeghi et al., 2021). Hinton et al. (2023) found that family-centered telehealth interventions in early childhood were most effective when conducted in natural environments and emphasized parent–child interaction.

Effective FCIs require professional guidance and training for parents (Akemoğlu & Meadan, 2018). Telehealth-based parent coaching has emerged as a viable method for providing this support. Parent coaching involves professionals teaching parents strategies through guided practice and feedback, enabling them to refine their skills (Sone et al., 2021). Research suggests that early intervention programs incorporating coaching are significantly more effective than those without it (Akemoğlu & Meadan, 2018; Douglas et al., 2020; Lee et al., 2022; Sawyer & Campbell, 2017; Sone et al., 2021; Tanner & Dounavi, 2020; Vismara et al., 2018). Given the importance of early parent–child interactions in ASD interventions, addressing fathers’ participation barriers through telehealth and online coaching can yield significant benefits for children, families, and society.

### The Purpose

The purpose of this study is to investigate the effectiveness of the Turkish Version of the Responsive Teaching (TV-RT) program applied as an online group intervention between autistic children and their fathers. To this end, answers were sought to the following research questions:

1. Does the TV-RT program applied as an online group intervention affect the interactional behaviors of fathers?
2. Does the TV-RT program applied as an online group intervention affect fathers’ abilities to use TV-RT strategies?
3. Does the TV-RT program applied as an online group intervention affect autistic children’s interactional behaviors?
4. Does the TV-RT program applied as an online group intervention affect the pivotal behaviors of autistic children?
5. Does the TV-RT program applied as an online group intervention affect the social interactions and autistic behaviors of children?
6. What are the opinions of the experimental group fathers as active participants and mothers as outsiders of autistic children?

## 2. Method

### 2.1. Experimental Design

This study used a pre-test–post-test control group experimental design (PSECD; Büyüköztürk, 2002). Participants were randomly assigned to experimental and control groups, and then pre-test data were collected from both groups. After the pre-test data were collected, only those in the experimental group received the experimental intervention, while the control group did not. Finally, post-test data were collected from both groups.

### 2.2. Participants

Autistic children in early childhood and their fathers participated in this study. The other study participants were the researcher who conducted the evaluation and implementation process and an independent coder who collected the reliability data. While the evaluation process of this study started with 48 participants, including 24 autistic children and their fathers, four father–child pairs dropped out of the study for various reasons, and the experimental process of the study was completed with 40 participants, including 20 autistic children and their fathers. All participating fathers gave their informed consent for inclusion before they and their children participated in this study. This study was conducted in accordance with the Declaration of Helsinki, and the protocol was approved by the Ethics Committee of Anadolu University Ethical Committee (Project identification code: 68/85 and the date of approval: 26 June 2022).

*Autistic children and their fathers.* In the research process, firstly, suitable child participants with the following prerequisites were considered: (a) being between 36 and 78 months, (b) having a diagnosis of ASD obtained from official institutions, (c) no additional diagnosis other than ASD, and (d) pivotal behavior scores of three or below according to an assessment of the Responsive Teaching–Pivotal Behavior Profile (RT-PBP). After identifying the children who met the prerequisites, the suitability of the father participants for the study was also assessed. Prerequisites sought for father participants were as follows: (a) not having participated in an interaction-based early childhood intervention program similar to TV-RT before, (b) the scores of the interactional behaviors assessed by the Maternal/Parent Behavior Rating Scale—Turkish Version (M/PBRS-TV), (c) declaring that he can use the Zoom Meeting application via phone, tablet or computer, and (d) willingness to participate in the study voluntarily.

To identify the father–child pairs participating in this study, an introductory letter was written explaining the purpose, content, duration, and prerequisites of the study. Then, the educational supervisors of a center in Konya Province that serves only autistic children were interviewed and asked to inform and guide the fathers who receive and have received services in their center about this study. In this process, all fathers who met the appropriate conditions were asked to be included in the list without any number limit. After the interviews, eligible participants were given two weeks to apply. Immediately after notifying the eligible participants that were on the list, a schedule was established and announced to the fathers on the list. The assessments were conducted at a special education and rehabilitation center in Konya. After completing all evaluation sessions, the first independent coder calculated inter-coder reliability. After consensus was reached, father–child pairs considered eligible to participate in this study were identified and informed. An envelope containing a consent form was delivered to the fathers who met the eligibility requirements, and they were asked to read it carefully, complete it, seal the envelope, and return it to the special education center where the evaluations were conducted. The consent form was given to the families. After identifying the fathers and children participating in the study, each father–child pair was assigned a number, and participants in the experimental and control groups were identified using block randomization (J. Kim & Shin, 2014). Table 1 presents a descriptive analysis of the levels of the autistic children in the experimental and control groups, their interactional performances, and the interactional levels of the fathers. Demographic information about the autistic children and their fathers, who constituted the experimental and control groups of this study, is presented in Table 2. According to the demographic information obtained through the personal information form, the average age of the autistic children in this study was 4 years and 6 months (54 months). Of the autistic children, 10% were girls and 90% were boys. The average age of the fathers of the autistic children was 36 years. Of the fathers, 5% graduated from secondary school, 25% from high school, 20% with an associate’s degree, 35% with a bachelor’s degree, and 15% with a master’s degree. Regarding the occupational information of the fathers, 20% of the fathers participating in this study were members of the police force (military–police), 30% were public officials (civil servants–teachers), and 50% were private sector/self-employed.

According to the data in Table 1 and Table 2, the levels of autistic children in the experimental and control groups, their levels of autism, their levels of exhibiting social interaction behaviors, their levels of exhibiting autistic interaction behaviors, their levels of interaction behaviors, and their pivotal behavioral profile levels are quite close to each other. Regarding fathers, the levels of the fathers’ quality interaction skills and use of parental strategies were quite similar in the context of the experimental and control groups. In addition to the descriptive data, independent samples t-test analysis was performed on the pre-test scores so that there was no difference in the dependent variable scores of the groups before the intervention regarding age—child (t(20): 0.49, *p*: 0.63), age—father (t(20): 1.80, *p*: 0.09), the Turkish-Adapted Autism Behavior Checklist (TA-ABC) (t(20): 0.36, *p*: 0.72), the Social Interaction Assessment Instrument—Turkish Version (SIAI-TV)–Typical Interaction Score (t(20): 0.04, *p*: 0.96), the Social Interaction Assessment Instrument—Turkish Version (SIAI-TV)–autistic interaction score (t(20): 0.81, *p*: 0.42), and the Child Behavior Rating Scale—Turkish Version (CBRS-TV)–total score (t(20): 0.79, *p*: 0.44); the Responsive Teaching–Pivotal Behavior Profile (RT-PBP)–cognitive development (t(20): 1.07, *p*: 0.30), the Responsive Teaching–Pivotal Behavior Profile (RT-PBP)–communication (t(20): 0.71, *p*: 0.49), the Parental/Maternal Behavior Rating Scale—Turkish Version (P/MBRS-TV)–Sensitivity–Responsiveness score (t(20): 0.69, *p*: 0.50), the Parental/Maternal Behavior Rating Scale—Turkish Version (P/MBRS-TV)–Emotional Expressiveness score (t(20): 1.53, *p*: 0.14), the Parental/Maternal Behavior Rating Scale—Turkish Version (P/MBRS-TV)–Directiveness score (t(20): 1.91, *p*: 0.07), the Responsive Teaching–Parent Strategy Profile (RT-PSP)–cognitive development score (t(20): 0.18, *p*: 0.86), and the Responsive Teaching–Parent Strategy Profile (RT-PSP)–communication development score (t(20): 0.32, *p*: 0.75).

*Implementer.* The implementer, the first author who conducted this study’s evaluation and implementation sessions, completed his undergraduate education in Special Education at Anadolu University in 2002 and his master’s degree in special education at Marmara University in 2013. The implementer had 22 years of experience working with autistic children and their families and had practitioner certificates of assessment tools and the TV-RT program and ETEÇOM. This study is part of the first author’s (the implementer) doctoral thesis under the second author’s supervision and the third author’s support.

*Coders.* Two independent coders took part in this study and collected inter-coder reliability and implementation reliability data by watching randomly selected video recordings from the data of all phases. The first independent coder completed his undergraduate and graduate education in Special Education. He had 18 years of experience working with autistic children and their families. The second independent coder completed his undergraduate and graduate education in Special Education. He had 15 years of experience working with children with developmental disabilities and their families. The independent coders have received training and application certificates for assessment tools and the TV-RT program.

### 2.3. Data Collection Tools

Various data collection instruments were used to assess multiple variables of the study participants. The personal information form was only completed at the pre-test and was administered to fathers in both the experimental and control groups. In addition to information about the fathers, the personal information form included demographic information about the mother and child. The Parental/Maternal Behavior Rating Scale—Turkish Version (P/MBRS-TV) and the Responsive Teaching–Parent Strategy Profile (RT-PSP) scales, which were used for the dependent variables of the study, were collected in both pre-test and post-test sessions and were administered to fathers in both the experimental and control groups. Finally, the satisfaction questionnaire, which constitutes the study’s social validity data, was administered only to the fathers in the experimental group and only in the post-test sessions.

Regarding the children, the Turkish-Adapted Autism Behavior Checklist (TA-ABC) was used only at the pre-test level to assess the level of ASD in the children. In contrast, the Social Interaction Assessment Instrument—Turkish Version (SIAI-TV), the Child Behavior Rating Scale—Turkish Version (CBRS-TV), and the Responsive Teaching–Pivotal Behavior Profile (RT-PBP) were used at the pre-test and post-test levels in both the experimental and control groups. Regarding the social validity of this study, satisfaction questionnaires were applied to fathers and mothers.

*Personal information form.* At the beginning of this study, a personal information form containing demographic information about the family and children was used. The form included demographic information about the mother, father, and child.

*Turkish-Adapted Autism Behavior Checklist (TA-ABC; Ozdemir et al., 2013).* To compare the levels of autistic children in the experimental and control groups and to determine whether there was a significant difference between the groups, the TA-ABC was used. The TA-ABC, originally known as the Autism Behavior Checklist, was adapted into Turkish by Ozdemir et al. (2013), and Turkish validity and reliability studies were conducted. In this tool, which contains 49 items, the first 42 items are administered to all children, while the remaining 7 items are administered additionally if the child has language skills. Cut-off scores are calculated separately for speaking and non-speaking children at the end of the assessment tool. Depending on the child’s score, the degree to which the child is affected by ASD and the level of support required can be determined. For speaking children, a score of 0–4 indicates “a very low likelihood of ASD”, 5–16 indicates “a mild need for support”, 17–25 indicates “a moderate need”, and 26–49 indicates “a high need for support”. Cut-off scores are different for children without language skills. For nonverbal autistic children, a score of 0–7 indicates “a very low likelihood of ASD”, a score of 8–21 indicates “a mild need for intervention”, a score of 22–28 indicates “a moderate need for intervention”, and a score of 29–42 indicates “a high need for intervention”.

*The Social Interaction Assessment Instrument—Turkish Version (SIAI-TV; Aksoy & Diken, 2016).* SIAI-TV was used to determine the effect of the intervention on children’s social interaction behaviors (typical social interaction and autistic interaction). Turkish validity and reliability studies of this comprehensive assessment set were conducted and adapted into Turkish by Aksoy and Diken (2016). The SIAI-TV assessment tool categorizes children’s spontaneous and adult-directed responses in 48 equal observation intervals. At the end of every 10 s, the child’s behavior was coded and assigned to categories such as interaction, independent play, unresponsiveness/nonreactivity, and negative aggression. At the end of the assessment, the number of observations in each category is calculated and converted to a standard score to obtain the child’s typical social interaction and autistic interaction scores. The sum of the scores for “independent play”, “unresponsiveness/nonreactivity”, and “negative aggressiveness” on the SIAI-TV gives the “autistic interaction” score, while the “interaction” score refers to the “social interaction” score. The KR-21 (Kuder–Richardson) value calculated for the internal reliability coefficient of the SIAI-TV was 0.83, and it is a test with high validity and reliability in analyzing children’s social interaction patterns (Aksoy & Diken, 2016).

*Child Behavior Rating Scale—Turkish Version (CBRS-TV; Ö. Diken, 2009).* The CRBS-TV assessed children’s interactional behaviors with their fathers in the experimental and control groups. The CRBS-TV was initially developed by Mahoney and Wheeden (1999) and the Turkish validity and reliability studies were completed by Ö. Diken (2009). The CRBS-TV consists of seven items under two subscales entitled Attention and Initiation. A five-point Likert-type rating (1 being “very low”, 3 being “medium”, and 5 being “very high”) is used for all items in the scale, which is used by observing or recording and analyzing children’s behavior during 10–15 min of free-play interaction between the adult and the child. A child’s interaction score of 5 points on both subscales indicates that the child is at the required level of interactional behavior. A total score is obtained from the scale with two subscale scores. The scale’s reliability for this study was calculated separately for each subscale and for the total scale score. The internal consistency was 0.98 in the pre-test and 0.98 in the post-test for the CRBS-TV Attention subscale,.87 in the pre-test and 0.97 in the post-test for the CRBS-TV Initiation subscale, and 0.96 in the pre-test and 0.99 in the post-test for the CRBS-TV total scale. Based on these results, the scale is a reliable measurement tool.

*The Responsive Teaching–Pivotal Behavior Profile (RT-PBP; İ. H. Diken, 2020).* The RT-PBP is an assessment form included in the Turkish version of the Responsive Teaching (TV-RT) program to determine children’s performance at the beginning of the implementation of pivotal behaviors and to measure and monitor their progress during the implementation process. To determine the acquisition level of the pivotal behaviors, videotapes of 10–15 min of free-play interaction between the adult and the child were used by observing, recording and analyzing the child’s behavior during the interaction. The RT-PBP consists of 15 items in three sub-dimensions: cognitive development, communicative development, and social–emotional development domains. Since social–emotional development domain strategies were not included in this study, the subscale related to this subdomain was not used. The cognitive development domain includes assessment tools related to five pivotal behaviors, such as “social play, initiating interaction, practicing, exploring, and problem-solving”, while the communication development domain also includes assessment tools related to five pivotal behaviors, such as “participation in activities, joint attention, vocalization, purposeful communication, and conversation”. A five-point Likert-type rating is used for all assessment tools; 1 means “very low”, 3 means “moderate”, and 5 means “very high. A score of 4 or above on the RT-PBP indicates that the child has acquired the relevant pivotal behavior. A total score for each domain is calculated as the sum of the five pivotal behaviors divided by five. In this study, the internal consistency coefficient for the reliability of the RT-PBP was calculated separately for each sub-domain. The Cronbach’s alpha values were 0.90 in the pre-test and 0.97 in the post-test for the RT-PBP–cognitive development, 0.92 in the pre-test, and 0.97 in the post-test for the RT-PBP–communication. Based on these results, the RT-PBP was found to be a reliable measurement tool.

*The Parental/Maternal Behavior Rating Scale—Turkish Version (P/MBRS-TV; Ö. Diken, 2009).* The P/MBRS-TV is a scale that assesses the interactional behaviors of adults/parents with their children (Ö. Diken, 2009). The P/MBRS-TV includes 12 parental behaviors significantly impacting children’s development (Mahoney, 2008). Consisting of three subscales, namely Sensitivity–Responsiveness, Emotional Expressiveness, and Directiveness, a five-point Likert-type rating (1 being “very low”, 3 being “medium”, and 5 being “very high”) is used for all items in the scale and scored by observing or recording and analyzing adults’ behavior during 10–15 min of free-play interaction between the adult and the child. An adult’s interaction score of 4 or 5 on the Sensitivity–Responsiveness and Emotional Expressiveness subscales indicates that the adult exhibits high-quality interactional behaviors. In contrast, a score of 3 on the Directiveness subscale demonstrates that the adult exhibits high-quality interactional behaviors. The internal consistency for the reliability of the scales was calculated separately for each subfactor. The internal consistencies (Cronbach’s alpha analysis) were 0.97 in the pre-test and 0.98 in the post-test for the P/MBRS-TV–Sensitivity–Responsiveness, 0.88 in the pre-test, and 0.95 in the post-test for the P/MBRS-TV–Emotional Expressiveness, and 0.87 in the pre-test and 0.94 in the post-test for the P/MBRS-TV–Directiveness. These results indicate that the scale is a reliable measurement tool.

*Responsive Teaching–Parent Strategy Profile (RT-PSP; İ. H. Diken, 2020).* The RT-PSP is an assessment form included in the TV-RT program to assess adults’ use of the TV-RT strategies. Videotapes of 10–15 min of free-play interaction between the adult and the child were used by observing, recording, and analyzing the adults’ use of TV-RT strategies during the interaction with their children. The RT-PSP consists of 15 items in three sub-dimensions: cognitive development, communicative development, and social–emotional development. Since social–emotional development strategies were not included in this study, the subscale related to this subdomain was not used. Cognitive development includes assessment tools related to five pivotal behaviors, such as social play, initiating interaction, practicing, exploring, and problem-solving, while communication development also includes assessment tools related to five pivotal behaviors, such as participation in activities, joint attention, vocalization, purposeful communication, and conversation. A five-point Likert-type rating is used for all assessment tools; 1 means “very low”, 3 means “moderate”, and 5 means “very high”. An adults’ score of 4 or above on the RT-PSP indicates that the adults use most of the RT-PSP strategies and shows high quality of interaction regarding related pivotal behavior. A total score for each domain is calculated as the sum of the five pivotal behaviors divided by five. In this study, the internal consistency coefficient for the reliability of the RT-PSP was calculated separately for each sub-domain. The Cronbach’s alpha values were 0.86 in the pre-test and 0.95 in the post-test for the RT-PSP–cognitive development, 0.89 in the pre-test and 0.97 in the post-test for the RT-PBP–communication. Based on these results, the RT-PBP was found to be a reliable measurement tool.

*Satisfaction Questionnaires.* While a seven-question satisfaction questionnaire was administered to fathers to obtain their opinions about the format and content of the Online Group TV-RT program, the satisfaction questionnaire administered to mothers included five questions about the impact of the program on the daily lives of fathers and children. In addition, the questionnaires also included an open-ended question such as “Please indicate your other opinions, if any, about the Online Group TV-RT program”. Both questionnaires were prepared as 5-point Likert scale (1 means “totally disagree”, 3 means “medium agree”, 5 means “strongly agree”). The satisfaction questionnaire was prepared by the researcher and finalized after receiving the opinions of three experts working in the field. The data obtained from the satisfaction questionnaire were part of the social validity of this study. Some of the questions for the fathers were “The Online Group TV-RT program was clear and understandable”, “I found the strategies explained in the Online Group TV-RT program useful”, “The Online Group TV-RT program had a positive impact on my child’s play and interaction behavior”, “I was satisfied with the content of the Online Group TV-RT program”, and “I would recommend the Online Group TV-RT program to other parents”, while some of the questions for the mothers were “The Online Group TV-RT program created positive changes in my husband’s play and interaction behavior”, “The Online Group TV-RT program created positive changes in my child’s play and interaction behaviors”, and “I am satisfied that my spouse participated in the Online Group TV-RT program”.

### 2.4. Independent Variable and Procedure

The independent variable of the study was the TV-RT program (İ. H. Diken, 2020), which was applied online as a group training for fathers with autistic children in early childhood. The RT was initially developed by Mahoney and MacDonald (2004), revised by Mahoney and Perales (2019) in the United States, and adapted into Turkish by İ. H. Diken (2013, 2020). The second version of the TV-RT (İ. H. Diken, 2020) is an intervention package that includes 15 pivotal behaviors and 63 interaction strategies in 3 developmental domains (cognitive, communication, and social–emotional development) and 109 discussion items. The purpose of the TV-RT is to improve the quality of the interactions of adults (especially parents) who spend most of their time with the target child during the family’s daily routines and directly impact the child’s development. Originally, as specified in its manual, specific strategies were defined for each pivotal behavior in each developmental domain, and strategies were introduced to adults verbally, and then positive and negative examples of strategies were modeled and coached in face-to-face sessions with the targeted child and his/her primary caregiver in home or clinical settings. The program suggests one or two weekly sessions, with one or two days off between sessions. However, this study adapted the TV-RT program to be used online with fathers. This study included 2 developmental areas (communication and cognitive development), 10 core behaviors, 43 interaction strategies, and 68 discussion points. The TV-RT sessions with fathers were conducted following the online TV-RT implementation guide. All sessions were conducted via Zoom Meeting Pro, and each session was recorded and uploaded to the Vimeo application. Fathers were able to access the video recordings via the link sent. The experimental intervention period lasted 6 weeks and 12 sessions, with 2 sessions per week. The sessions lasted 900 min, with the shortest session lasting 65 min in the first session and the longest lasting 91 min in the second session. The sessions lasted 75 min on average. In total, 43 strategies and 68 information notes on ten pivotal behaviors were shared. Table 3 shows the content and duration of each session and a summary of the program.

The sessions in the experimental intervention phase consisted of five stages. These stages were (1) preparation, (2) introduction, (3) providing information on the TV-RT discussion points and strategies, (4) monitoring–interpretation, and (5) finalization: (1) Preparation and controls were completed before the sessions. Before each session, it was checked whether the videos, ones sent by fathers and ones already in the TV-RT Automation System, were working and whether the documents to be shared were opened. The most crucial parts of the TV-RT strategies and information notes were highlighted and recorded. (2) The sessions started with a summary of the previous week. Fathers talked about what they remembered about the previous session, and the transfer of the information to practice was examined through the videos they sent. (3) The TV-RT discussion points and strategies related to that session were first projected on the screen as PDFs and then explained. (4) By showing pre-prepared training videos, the strategies covered in that session were explained and exemplified with the fathers’ practice videos recorded during their daily play or other routine practices with their children sent before each session and commented on. (5) The last part of the sessions consisted of going over the session in general terms, confirming what we remembered, and planning the next session. How strategies can be transferred to their children’s daily routines was also discussed, and suggestions were provided. Fathers were frequently given the right to speak at all these stages.

While the experimental group received intervention, fathers in the control group did not receive any intervention during the program. They were only involved with pre- and post-test procedures to collect the data. However, after the study was completed, a short (2 sessions) intervention was provided to fathers in the control group, and all materials (strategies and discussion points) were shared with them. During intervention, both autistic children groups received the same traditional (mostly Applied Behavior Analysis–ABA based) training at the same center from a private rehabilitation and special education center in Konya, while autistic children in the experimental group received indirect intervention by their fathers as part of the procedure.

### 2.5. Data Collection and Analysis

Data in the pre- and post-test phases were collected at a private special education and rehabilitation center in Konya, where the children were already receiving education. The room consisted of a table, two chairs, a carpet, toys suitable for interaction on the rug and the shelves next to it, a tripod, and a camera. After the researcher met with the father, who received preliminary information and had already provided consent to participate in the study, he summarized what would be carried out in the evaluation process, gave the estimated evaluation time, and answered any questions. All the evaluation sessions were videotaped. A single video recording was taken before and after the intervention. After the tripod, video camera, and environment were prepared, the fathers were told that a 10–15 min video recording would be taken and that they could interact with their children with the toys they wanted, either on the floor/carpet or at the table. They were told, “Please play with your child the way you always play at home”.

Data collected in this study were first checked for outliers and missing values, and erroneous data entries were controlled. In line with the results obtained by testing the analyses’ assumptions and normal distribution properties, it was determined that the data distribution exhibited a normal distribution, and parametric analyses (Covariance) were used. In the analyses, 5%, i.e., *p* ≤ 0.05, was accepted, and Bonferroni correction was applied; therefore, the significance level was adjusted to control the overall likelihood of a Type I error (false positive) for all Covariance analyses. Effect sizes (r = z/√*n*) were calculated for the analysis results in which significant differences were obtained. The η^2^ values were obtained; Cohen’s effect size interpretation (1988) was used. Accordingly, “0.1” was considered a small effect, “0.3” a medium effect, and “0.5 and above” a large effect.

Reliability data were collected with the blind coding principle. The data from different study phases were color-coded, and the external coders were not given any information about this. The coders coded the sessions without knowing which phase they belonged to. Thus, the risk of coder bias or expectations was eliminated. Inter-coder reliability data were collected for all effectiveness data in the study. Video recordings corresponding to 30% of the father and child evaluations in the pre-test and post-test sessions of the study were coded separately by two independent coders. The formula “Agreement/(Agreement + Disagreement) × 100” was used to calculate inter-coder reliability. As a result of the calculation, the reliability coefficients obtained in the pre-test and post-test were found to be 91% for the TV-P/MBRS, 93% for the TV-CBRS, 90% for the SIAI-TV, 91% for the RT-PBP, and 90% for the RT-PSP.

The first independent external coder collected the fidelity of the intervention to evaluate whether the online group TV-RT program was applied as an “online group TV-RT implementation guide”. The implementation fidelity form was introduced to the coder, and any questions were answered. While calculating the implementation reliability, the formula “(observed correct practitioner behavior/expected practitioner behavior) × 100” was used. The reliability coefficient obtained for the implementation reliability was calculated as 96%.

## 3. Results

### 3.1. Effect of the TV-RT Program on Fathers’ Interactional Behaviors

The results presented in Table 4 indicate a significant difference in the “Being Sensitive–Responsive” subscale of the P/MBRS-TV between the children of fathers who participated in the online group intervention of the TV-RT program and those who did not (F(1, 17) = 256.634, *p* < 0.05). Specifically, post-test scores of fathers who received the online TV-RT training (X = 4.47, SD = 0.78) were higher compared to those who did not receive the training (X = 2.23, SD = 0.90), with a large effect size (Partial Effect Size = 0.93). In contrast, the pre-test scores of the experimental group (X = 1.73, SD = 0.44) and the control group (X = 1.90, SD = 0.76) were considerably lower. Similarly, for the “Being Emotional–Expressive” subscale, a significant difference was observed between the groups (F(1, 17) = 165.280, *p* < 0.05), with the fathers who underwent the online group intervention scoring higher (X = 4.00, SD = 0.43)compared to those who did not receive the intervention (X = 2.70, SD = 0.67), again with a large effect size (Partial Effect Size = 0.90). Additionally, Table 4 shows that there was a significant difference in the “Being Achievement-Oriented and Directive” subscale of the P/MBRS-TV (F(1, 17) = 43.599, *p* < 0.05). Post-test results indicate that fathers in the experimental group (X = 2.77, SD = 0.39) scored higher than those in the control group (X = 1.87, SD = 0.69). The effect size for this difference was large (Partial Effect Size = 0.71). In other words, fathers in the experimental group showed significantly more sensitive, responsive, and emotionally expressive behaviors (e.g., acceptance, enjoyment, warmth) than the fathers in the control group. There were also more appropriate directive and achievement-oriented behaviors toward their autistic children than the fathers in the control group.

### 3.2. Effect of the TV-RT Program on Fathers’ Use of TV-RT Strategies

According to the findings in Table 5, significant differences were observed in the RT-PSP cognitive development scores between the fathers who received and those who did not receive the online TV-RT intervention (F(1, 17) = 61.524, *p* < 0.05). The experimental group (X = 4.17, SD = 0.55) showed higher post-test scores compared to the control group (X = 2.55, SD = 0.65), with a large effect size (Partial Effect Size = 0.78). A similar significant difference was noted for the RT-PSP communication scores (F(1, 17) = 46.985, *p* < 0.05). The fathers who participated in the intervention (X = 4.20, SD = 0.51) scored higher than those in the control group (X = 2.58, SD = 0.87), with a large effect size (Partial Effect Size = 0.73). In other words, the fathers in the experimental group showed significantly qualified interaction strategies, both supporting cognitive and communication development defined by the TV-RT with their autistic children compared to the fathers who did not receive the intervention.

### 3.3. The Effect of the TV-RT Program Applied as an Online Group Intervention on Interactional Behaviors of Autistic Children

The results presented in Table 6 indicate a significant difference in the CBRS-TV Initiation scores between children whose fathers participated in the online group intervention of the TV-RT program and those whose fathers did not (F(1, 17) = 89.940, *p* < 0.05). Specifically, descriptive data revealed that the post-test scores of the children in the experimental group (M = 4.20, SD = 0.50) were higher compared to those in the control group (M = 2.56, SD = 0.96). In contrast, the pre-test scores for the experimental group (M = 2.03, SD = 0.48) and the control group (M = 1.86, SD = 0.57) were similar. The effect size for this difference was large (Partial Effect Size = 0.84).

Similarly, as shown in Table 6, a significant difference was observed in the CBRS-TV Attention scores between the children of fathers who received the TV-RT online group intervention and those who did not (F(1, 17) = 94.164, *p* < 0.05). Descriptive data indicated that the post-test scores of children in the experimental group (M = 4.07, SD = 0.50) were significantly higher than those in the control group (M = 2.27, SD = 1.12). Pre-test scores for the experimental group (M = 1.82, SD = 0.44) and the control group (M = 1.62, SD = 0.81) were comparable. The effect size for this finding was also large (Partial Effect Size = 0.84).

Finally, according to the results in Table 6, a significant difference was found in the CBRS-TV total scores between children whose fathers participated in the TV-RT program and those whose fathers did not (F(1, 17) = 162.500, *p* < 0.05). Descriptive data demonstrated that the post-test scores for the children in the experimental group (M = 4.12, SD = 0.47) were higher than those of the control group (M = 2.41, SD = 1.02). The pre-test scores for the experimental group (M = 1.91, SD = 0.44) and the control group (M = 1.72, SD = 0.68) were similar. The effect size for this difference was high (Partial Effect Size = 0.90).

In other words, autistic children in the experimental group showed meaningfully higher Initiation, Attention, and total interactional behaviors than those in the control group. It meant they showed more interest toward actions and their fathers, initiated more interactions, and showed higher joint attention and cooperation and persistence than those in the control group.

### 3.4. The Effect of the TV-RT Program Applied as an Online Group Intervention on Pivotal Behaviors of Autistic Children

The findings presented in Table 7 indicate a significant difference in the RT-PBP cognitive development scores between the children of the fathers who participated in the online group intervention of the TV-RT program and those whose fathers did not (F(1, 17) = 32.500, *p* < 0.05). Specifically, descriptive data revealed that the post-test scores of children whose fathers received the intervention (M = 4.32, SD = 0.79) were significantly higher than the post-test scores of children whose fathers did not receive the intervention (M = 2.70, SD = 0.91). In contrast, the pre-test scores for the experimental group (M = 2.48, SD = 0.69) were slightly higher than those of the control group (M = 2.15, SD = 0.47). The effect size for this difference was large (Partial Effect Size = 0.65).

In addition, Table 7 shows a significant difference in the RT-PBP communication domain scores between the two groups of children (F(1, 17) = 150.893, *p* < 0.05). Descriptive data indicated that the post-test scores of the children whose fathers received the online group intervention (M = 4.20, SD = 0.51) were substantially higher than the post-test scores of children whose fathers did not receive the intervention (M = 2.58, SD = 0.87). The pre-test scores of the experimental group (M = 1.73, SD = 0.35) were slightly higher than those of the control group (M = 1.67, SD = 0.35). The effect size for this significant difference was also large (Partial Effect Size = 0.89). In other words, the autistic children in the experimental group showed meaningfully higher pivotal behaviors on social play, initiating interaction, practicing, exploring, and problem-solving regarding the cognitive development domain and on participation in activities, joint attention, vocalization, purposeful communication, and conversation regarding the communication development domain than the autistic children in the control group.

### 3.5. The Effects of the TV-RT Program Applied as an Online Group Intervention on Social Interaction Behaviors of Children

The results presented in Table 8 indicate a significant difference in the SIAI-TV Social Interaction scores between the children of the fathers who participated in the online group intervention of the TV-RT program and those whose fathers did not (F(1, 17) = 27.776, *p* < 0.05). Specifically, post-test scores for the children in the experimental group (M = 25.70, SD = 7.69) were notably higher than those in the control group (M = 18.20, SD = 7.16). In contrast, pre-test scores for both groups were comparable, with the experimental group scoring M = 13.80 (SD = 4.34) and the control group, M = 13.90 (SD = 6.38). The effect size for this difference was large (Partial Effect Size = 0.62).

Similarly, Table 8 shows a significant difference in the SIAI-TV autistic interaction scores between the children of fathers who received the online TV-RT intervention and those who did not (F(1, 17) = 58.967, *p* < 0.05). Descriptive data revealed that post-test scores for the experimental group (M = 26.10, SD = 11.60) were significantly lower compared to the control group (M = 34.70, SD = 12.50). Pre-test scores were M = 44.00 (SD = 11.60) for the experimental group and M = 40.00 (SD = 12.60) for the control group. The effect size for this difference was large (Partial Effect Size = 0.77). In other words, autistic children in the experimental group showed significant improvements in their autistic interaction behaviors and higher typical social interaction behaviors than those in the control group.

### 3.6. Fathers’ and Mothers’ (as Outsiders) Opinions on the TV-RT Program

The fathers who participated in the online group intervention of the TV-RT program were asked to provide feedback on various aspects of the program. The results indicate a strong positive reception toward the program. A vast majority of the participants (90%) agreed that the TV-RT program was clear and understandable, with 10% indicating a neutral stance. Regarding the subject mastery of the instructors, all fathers (100%) expressed satisfaction, agreeing that the facilitators had sufficient expertise to explain the program effectively. The usefulness of the strategies introduced in the program was highly rated, with 80% of the participants expressing agreement, while 20% were neutral. Additionally, when asked about the program’s impact on their interaction skills, 70% of fathers felt that the program significantly enhanced their abilities, with 30% remaining neutral. The program also had a notable effect on the children’s play and interaction behaviors. Half of the fathers (50%) reported a positive impact, while the other 50% expressed neutrality. When considering overall satisfaction, 80% of the fathers were satisfied with the content of the program, with 20% being neutral. Finally, the program was highly recommended by the participants, as all fathers (100%) indicated that they would suggest the TV-RT program to other parents. In summary, the feedback from the fathers indicates a strong approval of the TV-RT program, highlighting its clarity, usefulness, and positive impact on both their skills and their children’s behaviors.

There was also an open-ended question at the end of the satisfaction questionnaire. “Please write your opinions and suggestions about the TV-RT program applied as online group intervention here” was filled in by 8 out of 10 fathers. Fathers also stated high satisfaction with their statements. For example, Father 1 stated that *“First of all, thank you very much. He taught us how to play games with our child. He taught us how to teach the child the meanings of daily life through play or how to continue the education process in the most effective way. The program taught us that what we think is right in child education is actually wrong and difficult ways. In other words, I can say that it is a very effective and contributing program”*. Father 4 emphasized that *“The TV-RT program is a program that awakens fathers to fully interact with their children, so every father should participate”.* Father 6 stated that *“God bless those who contributed”*. Father 8 stated that “*This training was a turning point in my life. I realized what I can do for my child’s development. I will be able to better interpret teachers’ practices”*, and lastly, Father 10 stated that “*I definitely think that all parents should receive these trainings. Thanks to this program, I have learned that there are many common misconceptions. My perspective on my child, my ability to understand him/her, and my ability to enter his/her world have increased”.*

After the completion of the TV-RT program applied as an online group intervention, a satisfaction questionnaire was also administered to the mothers of 10 children in the experimental group. The mothers’ feedback on the TV-RT program, delivered as an online group intervention, reveals generally positive responses across various aspects of the program. A significant majority (80%) of the mothers agreed that the program led to positive changes in their husbands’ play and interaction behaviors, with only a small proportion of participants (20%) expressing complete agreement. Regarding the impact of the program on their children’s play and interaction behaviors, 70% of mothers reported that the program created a positive change, while 20% expressed a strong agreement, and 10% remained neutral. When asked about the frequency of interaction between fathers and children, 70% of the mothers indicated that the program contributed to an increase in interaction frequency, while 30% reported a neutral stance.

The program’s acceptance among participants was also high, as 90% of the mothers expressed satisfaction that their husbands participated in the intervention, with only 10% being neutral. Additionally, 90% of the mothers would recommend the TV-RT program to other parents, further emphasizing the program’s positive reception. Overall, the mothers’ opinions highlight the effectiveness of the TV-RT program in enhancing family interactions, with 80% of the responses reflecting strong agreement regarding the program’s positive impact.

There was also an open-ended question at the end of the satisfaction survey. Five out of ten mothers filled in the item “Please write your opinions and suggestions about the TV-RT program applied as an online group intervention here”. Mothers also stated high satisfaction with their statements. For example, Mother 1 stated that *“First of all, as the mother of a child with autism, I would like to express my sincere thanks to the implementer. My husband used to play games with my child, but after my husband started working with the implementer, these games gained meaning…”*. Mother 3 stated that *“Very nice program, thank you…”*. Mother 4 emphasized that “*The activities and games that other participants also did gave my husband ideas… We were very pleased, it was a useful activity*”. Mother 7 stated that “*my husband’s social skills of play, conversation, and interaction have improved a lot… A useful training… I recommend it to everyone”*. Lastly, Mother 10 stated that *“it is very nice to think especially for fathers… We also want it for mothers…”*

## 4. Discussion

This study examined the effectiveness of the Turkish Version of Responsive Teaching (TV-RT) as an online group intervention for preschool autistic children and their fathers. The findings align with the previous literature and offer insights into father participation, interaction behaviors, and the benefits of online implementation.

*Father Involvement.* The TV-RT program has been extensively studied with parents of autistic children (Mahoney et al., 2014; Alquraini et al., 2018; Selimoğlu & Özdemir, 2018; Toper et al., 2019). However, no prior research specifically targeted fathers, despite studies across different cultures (Turkey, South Korea, and the USA). Research on early intervention for fathers remains limited (Bendixen et al., 2011; Donaldson et al., 2011; Elder et al., 2011). Studies focusing on fathers and autistic children’s social interaction skills through online training are rare (Vismara et al., 2018; Yetkin, 2023). The limited involvement of fathers may stem from work schedules, time constraints, and researcher concerns about attrition (Gonzalez et al., 2023). Additional barriers include rigid training schedules and high expectations from experts (Klein et al., 2023; Marsack-Topolewski, 2023).

To enhance father participation, this study adopted flexible scheduling, considering personal availability. Sessions were spaced out to avoid conflicts with major events, such as sports competitions. Fathers with unpredictable schedules were provided recorded sessions to reduce barriers. Engagement was encouraged by discussing their experiences and expectations, while a WhatsApp group was used for reminders. Praise and encouragement in early sessions motivated participation, and mandatory camera usage enhanced interaction.

*Fathers’ Quality Interaction Behaviors*. Baseline assessments revealed that fathers exhibited directive and achievement-oriented behaviors while displaying low levels of sensitive and responsive interactions with their autistic children. This pattern aligns with prior studies on father–child interactions (Arslan & Diken, 2020; Flippin & Crais, 2011; Karaaslan, 2016; Ostfeld-Etzion et al., 2016). Through the TV-RT intervention, fathers developed more sensitive, emotionally expressive behaviors, which gradually replaced their directive approach. Weekly video analyses demonstrated an increase in responsive interactions, aligning with previous findings that emphasize the importance of parent–child interaction (Selimoğlu & Özdemir, 2018; Karaaslan et al., 2011; Mahoney & Perales, 2003; Toper et al., 2019).

*Children’s Interaction and Pivotal Behaviors*. Initially, there were no significant differences in social interaction levels between the children in the experimental and control groups. However, after the TV-RT intervention, the children whose fathers participated showed increased interactional behaviors, whereas those in the control group showed no notable change. This outcome is consistent with research indicating that parental improvements in interaction positively influence children’s social skills (Alquraini et al., 2018; Selimoğlu & Özdemir, 2018; Karaaslan et al., 2011, 2013; J.-M. Kim & Mahoney, 2004; Toper et al., 2019). Post-intervention assessments showed a transition from autistic social interaction patterns to more typical interactions in the experimental group, unlike the control group. This supports the notion that improved parent–child interaction fosters positive social development (Selimoğlu & Özdemir, 2018).

*Online Implementation*. Most father-involvement studies have traditionally been conducted face-to-face (Bendixen et al., 2011; Donaldson et al., 2011; Elder et al., 2011). However, recent studies highlight the necessity of online interventions in exceptional circumstances such as pandemics or natural disasters (Hinton et al., 2023). Remote interventions have gained traction as an accessible means to support families (Akemoğlu et al., 2022; Meadan & Daczewitz, 2015). This study’s online implementation yielded results comparable to in-person TV-RT applications (Selimoğlu & Özdemir, 2018; Karaaslan et al., 2011, 2013; Mahoney & Perales, 2003; Toper et al., 2019).

*Group Training*. Studies suggest that both individual- and group-based parent-mediated interventions can be effective (Garnett et al., 2022). However, group training is noted for its time- and cost-efficiency, as well as its potential to enhance learning through peer modeling (Vismara et al., 2018). Fathers in this study reported that group sessions reduced anxiety and facilitated learning. Some noted that observing other participants’ strategies and receiving collective feedback was valuable. Fathers appreciated the diverse perspectives provided in group training, reinforcing findings from prior research (Gonzalez et al., 2023; Klein et al., 2023; Marsack-Topolewski, 2023).

*Social Validity*. Post-intervention satisfaction surveys indicated that both fathers and mothers viewed the program positively. Fathers reported enhanced interactions with their children, increased understanding, and improved confidence, which translated into better family dynamics. These findings align with previous TV-RT studies (Selimoğlu & Özdemir, 2018; Toper et al., 2019). Notably, mothers, though not direct participants, reported benefiting from increased father–child engagement. Social validity data from non-intervention mothers are rare in the existing literature but provide valuable insights.

Research suggests a reciprocal relationship between parental perceptions of disability and their behavior (Kissel & Nelson, 2016). This study highlights the psychological and behavioral interplay between parents and children (Baker-Ericzén et al., 2005; Doubet, 2012; Falk et al., 2014; Gau et al., 2012; Ostfeld-Etzion et al., 2016; Ozturk et al., 2014). Addressing these psychological aspects in future interventions is recommended.

## 5. Conclusions, Limitations, and Suggestions

The increasing adoption of online family-centered interventions for fathers underscores the relevance of this study. By incorporating group training, parent coaching, and delayed feedback, the research offers a replicable model. Additionally, obtaining feedback from non-participating mothers adds originality to the findings.

Parental interaction training is essential as a preventative measure. Qualified parental behaviors can reduce children’s maladaptive behaviors, emphasizing the importance of widespread access to such programs. Expanding collaboration between government institutions, NGOs, and universities can enhance accessibility.

Despite the emphasis on father involvement in interventions, participation remains suboptimal. Future studies should explore strategies to enhance engagement, such as flexible scheduling and motivational incentives. The integration of mobile and web-based applications could further optimize accessibility (Akemoğlu et al., 2022; Hinton et al., 2023; Meadan & Daczewitz, 2015).

Several limitations should be considered. Cultural variations in parenting and child-rearing should be examined in future studies (Alquraini et al., 2018; Karaaslan et al., 2011; Mahoney & Perales, 2003; Selimoğlu & Özdemir, 2018; Toper et al., 2019; Töret et al., 2015). This study’s findings are based on a limited sample from Konya, Turkey, making generalization difficult. Larger, more diverse samples across different Turkish regions are recommended. Additionally, due to the study’s timeframe constraints, no longitudinal follow-up was conducted. Future research should assess the long-term impact of TV-RT interventions.

Further comparative research could evaluate the effects of father- versus mother-only interventions and compare online versus in-person approaches. Investigating the psychological impact of parent-mediated interventions in more detail would also be beneficial. Expanding similar studies to children with other developmental disabilities and their families would enhance the generalizability of findings.

This study contributes to the growing body of evidence supporting father-inclusive, online, family-centered interventions. By addressing the challenges of father participation and leveraging digital tools, future research can refine and expand effective intervention strategies for autistic children and their families.

## Figures and Tables

**Table 1:** Descriptive characteristics of participants.

		Experimental Group			Control Group		
		X	Min.	Max.	X	Min.	Max.
**Children** **Child**	TA-ABC * (Total Score)	20.80	15	28	20.00	10	32
	SIAI-TV ** (Typical Interaction Score)	13.80	6	20	13.90	4	24
	SIAI-TV (Autistic Interaction Score)	44.00	30	68	40.00	24	62
	CBRS-TV *** (Total score)	1.91	1	2	1.72	1	3
	RT-PBP **** (Cognitive Development Score)	2.48	1	2	2.15	1	3
	RT-PBP (Communication Development Score)	1.73	1	2	1.67	1	3
**Fathers**	M/PBRS-TV- ***** (Sensitivity–Responsivity Score)	1.73	1	2	1.90	1	3
	M/PBRS-TV- (Emotional Expressiveness Score)	1.66	1	2	1.96	1	2.6
	M/PBRS-TV- (Being Directiveness Score)	1.33	1	2	1.67	1	2
	RT-PSP ****** (Cognitive Development Score)	1.60	1	2	1.60	1	2
	RT-PSP (Communication Development Score)	1.73	1	2	1.67	1	2

* TA-ABC: Turkish-Adapted Autism Behavior Checklist; ** SIAI-TV: Social Interaction Assessment Instrument—Turkish Version; *** CBRS-TV: Child Behavior Rating Scale—Turkish Version; **** RT-PBP: Responsive Teaching–Pivotal Behavior Profile; ***** M/PBRS-TV: Maternal/Parent Behavior Rating Scale—Turkish Version; ****** RT-PSP: Responsive Teaching–Parent Strategy Profile.

**Table 2:** Demographic information of the participants in the study.

	No.	Child Age	Child Gender	Father Age	Father Education	Father Profession
**Experimental Group**	1	44 months	Male	40	Master’s Degree	CNC Operator
	2	45 months	Male	44	License	Air Chief Petty Officer
	3	69 months	Male	32	Associate Degree	Headmistress
	4	46 months	Male	39	High School	Director
	5	51 months	Male	39	License	Officer
	6	61 months	Male	41	Associate Degree	Gendarmerie
	7	78 months	Male	40	License	Teacher
	8	49 months	Male	41	Middle School	Free
	9	44 months	Male	32	High School	Warehouseman
	10	68 months	Female	29	High School	Cook
**Control Group**	11	66 months	Male	29	License	Cargo Employee
	12	45 months	Male	34	Master’s Degree	Teacher
	13	64 months	Male	39	Master’s Degree	Officer
	14	68 months	Male	37	Associate Degree	Free
	15	78 months	Male	35	License	Salesperson
	16	41 months	Male	27	High School	Body Shop
	17	37 months	Male	36	License	Military Personnel
	18	42 months	Male	31	Associate Degree	Free
	19	44 months	Male	38	License	Officer
	20	43 months	Female	38	High School	Security Officer

**Table 3:** TV-RT online sessions’ summary.

	Sessions	Strategies *	Discussion Points *	Duration of Training
	Session 1	“What is the TV-RT?” presentation		65 min
	Session 2	1-2-3	1-2-3-4	91 min
	Session 3	4-5-6-7	5-6-7-8-9	78 min
	Session 4	8-9-10-11	10-11-12-13-14	75 min
	Session 5	12-13-14-15	15-16-17-18-19-20	70 min
	Session 6	16-17-18-19	21-22-23-24-25-26	72 min
	Session 7	20-21-22-23	27-28-29-30-31-32	74 min
	Session 8	24-25-26-27	33-34-35-36-37-38	78 min
	Session 9	28-29-30-31	39-40-41-42-43-44-45	77 min
	Session 10	32-33-34-35	46-47-48-49-50-51-52	75 min
	Session 11	36-37-38-39	53-54-55-56-57-58-59	70 min
	Session 12	40-41-42-43	60-61-62-63-64-65-66-67-68	75 min
Total	12 sessions	43 strategies	68 discussion points	900 min

* Numbers indicate TV-RT strategy and discussion points’ number as stated in the TV-RT manual.

**Table 4:** Covariance results of the P/MBRS-TV subscales.

Source of Variance	Sum of Squares	Sd	Mean Squares	F	*p*	Partial Effect Size
Being Sensitive–Responsive Subscale						
Pre-test	10.889	1	10.889	93.820		
Group	29.787	1	29.787	256.634	0.001	0.93
Error	1.973	17	0.116			
Total	38.175	19				
Being Emotionally Expressive Subscale						
Pre-test	4.496	1	4.496	63.490		
Group	11.705	1	11.705	165.280	0.001	0.90
Error	1.204	17	0.071			
Total	14.150	19				
Being Achievement Oriented and Directive Subscale						
Pre-test	3.115	1	3.115	21.212		
Group	6.402	1	6.402	43.599	0.001	0.71
Error	2.496	17	0.147			
Total	9.661	19				

**Table 5:** Covariance results of the RT-PSP domain scores.

Source of Variance	Sum of Squares	Sd	Squares Average	F	*p*	Partial Effect Size
Cognitive Domain Score						
Pre-test	2.958	1	2.958	13.784		
Group	13.203	1	13.203	61.524	0.001	0.78
Error	3.648	17	0.215			
Total	19.809	19				
Communication Domain Score						
Pre-test	4.992	1	4.992	20.586		
Group	11.393	1	11.393	46.985	0.001	0.73
Error	4.122	17	0.242			
Total	22.182	19				

**Table 6:** Covariance results of the CBRS-TV.

Source of Variance	Sum of Squares	Sd	Mean Squares	F	*p*	Partial Effect Size
Initiation Score						
Pre-test	8.889	1	8.889	82.430		
Group	9.699	1	9.699	89.940	0.001	0.84
Error	1.833	17	0.108			
Total	24.061	19				
Attention Score						
Pre-test	11.562	1	11.562	92.483		
Group	11.772	1	11.772	94.164	0.001	0.84
Error	2.125	17	0.125			
Total	29.887	19				
Total Score						
Pre-test	10.324	1	10.324	159.449		
Group	10.521	1	10.521	162.500	0.001	0.90
Error	1.101	17	0.065			
Total	26.118	19				

**Table 7:** Covariance results of the RT-PBP.

Source of Variance	Sum of Squares	Sd	Mean Squares	F	*p*	Partial Effect Size
Cognitive Domain Score						
Pre-test	9.583	1	9.583	46.724		
Group	6.666	1	6.666	32.500	0.001	0.65
Error	3.487	17	0.205			
Total	26.138	19				
Communication Domain Score						
Pre-test	16.228	1	16.228	261.871		
Group	9.351	1	9.351	150.893	0.001	0.89
Error	1.053	17	0.062			
Total	28.159	19				

**Table 8:** Covariance results of the SIAI-TV.

Source of Variance	Sum of Squares	Sd	Mean Squares	F	*p*	Partial Effect Size
Social Interaction Score						
Pre-test	815.871	1	815.871	77.995		
Group	290.548	1	290.548	27.776	0.001	0.62
Error	177.829	17	10.461			
Total	1274.950	19				
Autistic Interaction Score						
Pre-test	2405.029	1	2405.029	189.310		
Group	749.128	1	749.128	58.967	0.001	0.77
Error	215.971	17	12.704			
Total	2990.80	19				

## Data Availability

Data is unavailable due to privacy or ethical restrictions approved by Anadolu University.
